# Autophagy at the intersection of aging, senescence, and cancer

**DOI:** 10.1002/1878-0261.13269

**Published:** 2022-07-09

**Authors:** Liam D. Cassidy, Masashi Narita

**Affiliations:** ^1^ Cancer Research UK Cambridge Institute University of Cambridge UK; ^2^ Tokyo Tech World Research Hub Initiative (WRHI), Institute of Innovative Research Tokyo Institute of Technology Yokohama Japan

**Keywords:** aging, autophagy, cancer, senescence

## Abstract

Autophagy is an evolutionarily conserved cellular process in which macromolecules undergo lysosomal degradation. It fulfills essential roles in quality controlling cellular constituents and in energy homeostasis. Basal autophagy is also widely accepted to provide a protective role in aging and aging‐related disorders, and its decline with age might precipitate the onset of a variety of diseases. In this review, we discuss the role of basal autophagy in maintaining homeostasis, in part through the maintenance of stem cell populations and the prevention of cellular senescence. We also consider how stress‐induced senescence, for example, during oncogene activation and in premalignant disease, might rely on autophagy, and the possibility that the age‐associated decline in autophagy might promote tumour development through a variety of mechanisms. Ultimately, evidence suggests that autophagy is required for malignant cancer progression in a number of settings. Thus, autophagy appears to be tumour‐suppressive during the early stages of tumorigenesis and tumour‐promoting at later stages.

## Graphical abstract

In this review, we discuss the role of basal autophagy in homeostasis through the maintenance of stem cell populations and the prevention of cellular senescence. We discuss how senescence, for example, during oncogene activation and in premalignant disease, may rely on autophagy, and how the age‐associated decline in autophagy could promote tumour development.

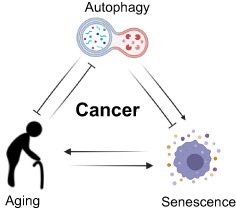

**Abbreviations**
AMPK: AMP‐activated protein kinase
APOE: apolipoprotein E
ATG5: autophagy‐related gene 5
ATG7: autophagy‐related gene 7
ATG8: autophagy‐related gene 8
BCL2: B‐cell lymphoma 2
BECN1: Beclin‐1
BUBR1: BUB1 mitotic checkpoint serine/threonine kinase B
CCFs: cytoplasmic chromatin fragments
CDK: cyclin dependent kinase
DNMT1: DNA methyltransferase 1
eIF5A: eukaryotic translation initiation factor 5A‐1
FOXO: forkhead box protein O
GATA4: GATA binding protein 4
HSC: hematopoietic stem cell
HSC70: heat shock cognate 71 kDa protein
IL6: interleukin 6
IL8: interleukin 8
LAMP2A: lysosome‐associated membrane protein 2, variant A
LC3: microtubule‐associated proteins 1A/1B light chain 3B
mTORC1: mammalian target of rapamycin complex 1
p62/SQSTM1: sequestosome‐1
PAX7: paired box protein Pax‐7
SAHF: senescence associated heterochromatin fragments
SASP: senescence associated secretory phenotype
SA‐β‐gal: senescence associated Beta‐galactosidase
SIRT1: sirtuin 1
TFEB: transcription factor EB

## 1 Introduction

Autophagy is a highly conserved process in which cellular components – from lipids and proteins, to large aggregates and organelles – are delivered to lysosomes for degradation. Autophagy was initially considered to be a broad non‐selective process, partly because autophagic activity can be dramatically upregulated during times of nutrient and of other cellular stresses, when intracellular components are mobilized and broken down to maintain energy homeostasis. It is now clear that this nutrient stress‐induced engagement concealed the remarkable complexity that exists with regard to cargo selection [1].

**Fig. 1:**
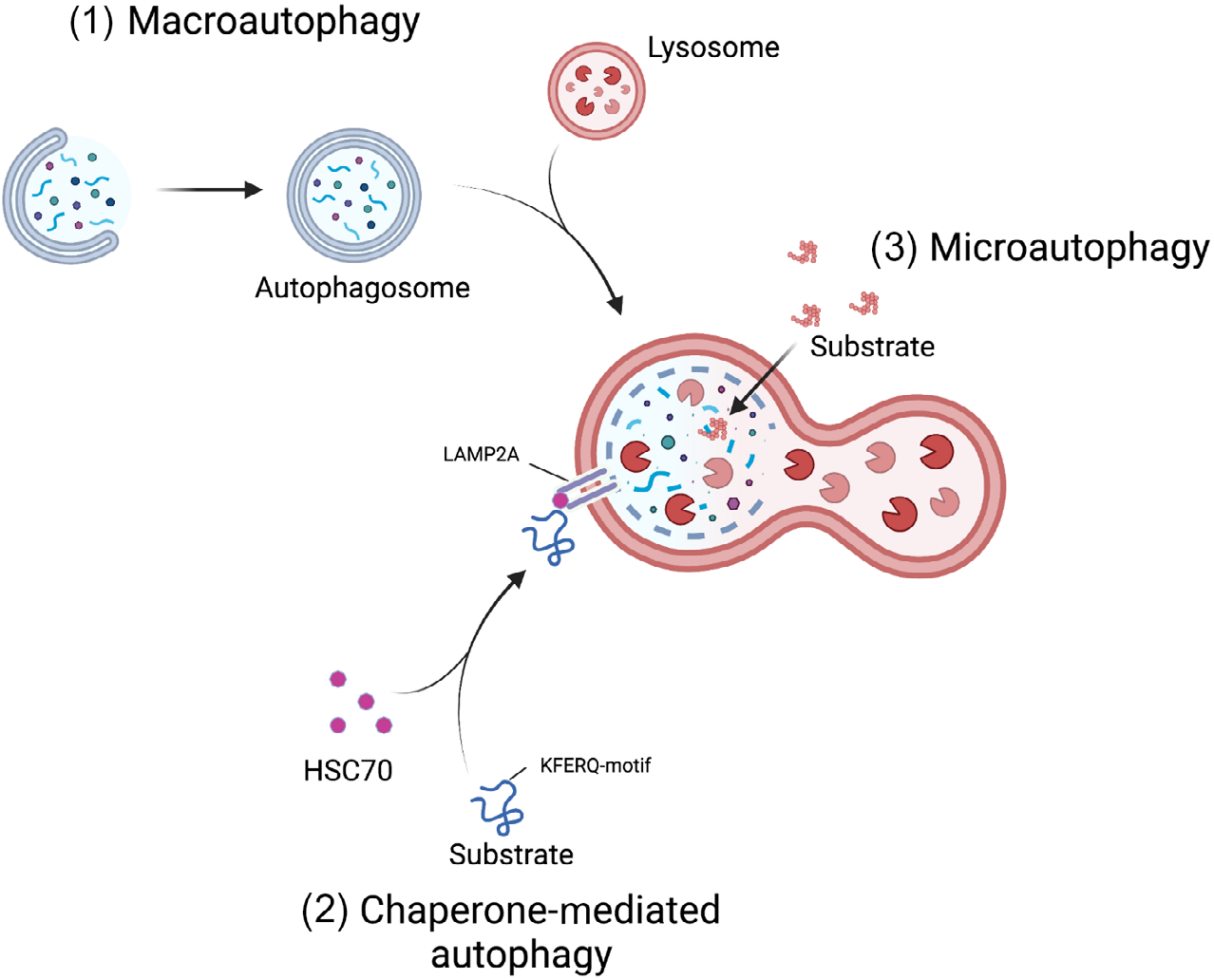
Three subtypes of autophagy. (1) During macroautophagy, cellular components are sequestered and isolated into a double‐membrane‐bound vesicle before fusing with a lysosome, forming an autolysosome, and leading to the breakdown of the vesicle contents. (2) During chaperone‐mediated autophagy proteins with a KFERQ‐like targeting motif are bound by the chaperone HSC70. The substrate‐HSC70 complex binds to the lysosomal membrane protein LAMP2A resulting in the translocation of proteins across the lysosomal membrane where they are subsequently degraded. (3) During microautophagy, cellular material is directly transported to late endosomes or directly to the lysosome for degradation through invagination of the vesicle membranes.

In addition to this bulk versus selective paradigm shift, we have also come to appreciate that autophagy is not simply one process in mammals, but three distinct processes (Fig. 1): macroautophagy, chaperone mediated autophagy, microautophagy. During macroautophagy, cellular components are sequestered and isolated into a double‐membrane‐bound vesicle (the autophagosome) before fusing with a lysosome, forming an autolysosome, leading to the breakdown of the vesicle contents. Chaperone‐mediated autophagy is a process in which proteins with a KFERQ‐like targeting motif can be recognized by the chaperone HSC70 before subsequent binding to the lysosomal membrane protein LAMP2A. This binding and ensuing formation of a translocation complex enable the transport of proteins across the lysosomal membrane where they are subsequently degraded [2]. Finally, during microautophagy cellular material is directly transported to the lysosome without the need for autophagosome biogenesis. Instead, late‐endosomes, or lysosomes, directly engulf cellular components before they are eventually broken down [3].

As the bulk of research has centred on macroautophagy, the term macroautophagy and autophagy are used interchangeably. However, significant evidence is accumulating that chaperone‐mediated autophagy has equally important implications in health and disease with regards to aging and cancer, and one must assume that the same is true of microautophagy. Here we focus primarily on macroautophagy, referring to it as simply autophagy due to its greater research focus in the community, however, our knowledge of the interplay of the three autophagic processes, and how they act in concert is poorly understood. If we are to understand the complex role autophagy has in mammalian cancer and aging, we are surely going to need to better balance our perspective on this.

In this review, we take a critical look at the data that underpins our understanding of when autophagy is modulated during aging and in the context of senescence and cancer. There is a complex interplay between these processes, autophagy is altered in aging, senescence, and cancer, equally its modulation can affect all three. In particular, we discuss the evidence that autophagy modulation through dietary, pharmalological, or genetic means from mammalian models may affect health and life span with a particular focus on stem cell functionality and cellular senescence. Simultaneously, we also place this information into the context of tumorigenesis where autophagy reactivation commonly occurs and is believed to promote tumor cell fitness. Finally, we argue that despite the clear links that exist between autophagy in aging, senescence, and in tumor development, our understanding of how altered autophagic flux influences these processes remains unclear. To ascertain this, we need more granular fundamental information about how and when autophagic flux changes, as well as more nuanced models that recapitulate these alterations, as opposed to blanket statements and an over‐reliance on complete knockout models.

## 2 Autophagy and aging

A central paradigm of both aging and autophagy research is that, with increasing age, there is a corresponding decrease in the rate of autophagic activity, at least in some tissues [4]. As autophagy is at the nexus of protein homeostasis, organelle turnover, and cellular metabolism, it is unsurprising that a swathe of evidence exists across species, both genetic and pharmacological, highlighting how the loss of autophagy is enough to drive cellular, tissue, and organismal dysfunction with detrimental effects on health and lifespan; by contrast boosting, autophagy has the opposite effects [5, 6, 7].

### 2.1 Lessons from model organisms

Historically some of the earliest data is leveraged from studies in lower organisms where the loss of autophagy has been shown to decrease life span and health span [8, 9], whilst overexpression of the autophagy machinery has been shown to promote longevity in some cases [10, 11]. Additionally, genetic perturbations that result in life‐span extension, also known as long‐lived mutants, have been found to be dependent on an intact autophagy system [12, 13, 14]. As loss of autophagy through genetic models or RNAi has been shown to reverse these longevity phenotypes in both *Caenorhabditis elegans* and *Drosophila melanogaster*, it suggests that autophagy may act as a central process in longevity across species [15, 16].

Traditionally, it has been a challenge to study the effects of systemic autophagy loss on longevity in mammalian models, because of the neonatal lethality that occurs in constitutive knockout models of key autophagy genes, and the rapid, severe, neurological phenotypes encountered upon whole‐body ablation of autophagy in the adult mouse [17, 18]. Consequently, much of the available evidence has been limited to tissue‐specific knockouts of essential autophagy genes. However, these are also complicated as the resultant phenotypes are often an amalgamation of both developmental and homeostasis effects. Recently, two models wherein autophagic activity is constitutively higher throughout life leading to health‐ and life‐span extension [19, 20], and two models wherein autophagy can be dynamically inhibited and restored [21], have provided fresh evidence that in the mammalian setting, autophagy acts causally at the interface with longevity.

Despite recent advances in our understanding of autophagy, our knowledge of how it is perturbed during aging and in disease states, particularly in cancer development, remains rudimentary. In *C. elegans* expression of a tandem reporter (Box 1) enabling autophagic activity to be determined not only confirmed that autophagic flux decreased upon entering old age, but uncovered evidence of tissue‐specific and differential age‐associated trajectories of autophagy activity, suggesting hitherto underappreciated spatial and temporal kinetics [22]. Further reinforcing the notion that we are still yet to truly understand how autophagy perturbations impact upon health and disease across the life course, in post‐reproductive *C. elegans*, life‐span can be extended through the inhibition of genes involved in the early stages of autophagy [23]. This seemingly paradoxical result is due to the alleviation of age‐associated dysfunctional autophagy which would otherwise result in neurodegeneration and shorten organismal life span [23].

Box 1 **Glossary**

Atg8/LC3 tandem reporters

Tandem LC3 reporters exploit the change in pH that accompanies lysosomal fusion with autophagosomes, and the different pH stability of fluorophores, e.g., GFP and mRFP. LC3 puncta mark autophagosome formation, in this case being both GFP and mRFP positive. However, during fusion with lysosomes, the GFP signal is quenched and only the acid‐resistant mRFP can be visualized. As such, the ratio of GFP/RFP positive, and RFP positive alone enables a more quantitative analysis of autophagic flux.

In mammalian models, tandem Atg8/LC3 reporter mice [18, 24, 25, 26, 27] have also been developed and helped to deconvolute the hierarchy of autophagic regulation by AMPK‐mTORC1 signaling the kidney [24], as well as improving our understanding the role of autophagy and its key modulators during reperfusion injury in the kidney and heart [25, 26, 27]. It appears that selective forms of autophagy also display tissue and age‐associated complexity in mammalian systems. This at least appears the case for mitophagy wherein single or tandem tagged mitochondria have been used to uncover developmentally regulated mitophagy processes and in cell‐type‐specific activities [28]. A reduction in mitophagy can also be found in particular regions of the brain with aging as well as in Parkinson's disease, Huntington's disease, and as a result of a high‐fat diet [29, 30]. However, whilst these data are insightful, there is limited data on pan‐tissue analysis in wild‐type, or genetically perturbed mammalian models, during natural aging, with age‐related disease states, or during different stages of cancer development.

It has been noted that a number of autophagy and lysosomal‐associated genes and proteins decrease with age [10, 31, 32, 33, 34], and that the increased expression of autophagy/lysosomal master regulator transcription factors, such as TFEB and FOXO, can promote health‐ and life‐span in an autophagy‐dependent manner [35, 36, 37]. Interestingly, Zhang et al. [38] reported that the TFEB protein expression is maintained by the unique hypusinated translation factor eIF5A and that this post‐translational modification of eIF5A is mediated by an endogenous polyamine spermidine. Antiaging and autophagy‐inducing effects of spermidine (or other natural polyamines) and age‐associated decline of the natural polyamines have been noted: the antiaging effect of spermidine appears to be autophagy‐dependent [39]. Furthermore, the reported negative regulator of autophagy, Rubicon, is increased in aged tissue across species and its reduction is associated with life‐span extension in *C. elegans* and *D. melanogaster*, as well as the amelioration of aging phenotypes in mice [40]. Although the entire picture is still vague, these studies provide some mechanistic insights into the age‐associated autophagy decline.

### 2.2 Autophagy and stem cells

Several reports have, nonetheless, causally linked a reduction in autophagy with aging through a reduction in stem cell capacity. Skeletal muscle stem cells (also known as satellite cells) from geriatric humans and mice display heightened levels of the autophagy adaptor p62/Sqstm1, and reduced autophagic flux in comparison to satellite cells isolated from young counterparts, correlating with a decrease in function and the induction of senescence [41]. This loss of functionality is purportedly linked in part to organelle quality control (specifically mitochondria) and genetic or pharmacological promotion of autophagy in geriatric satellite cells appears to restore functionality to some degree [41]. Furthermore, the loss of autophagy from genetically engineered mouse models leads to the disruption of both muscle fiber structure, function, and satellite cell number supporting a causal role of autophagy in maintaining tissue and cellular functionality [21, 41, 42].

Additionally, autophagy seems to have a role in hematopoietic stem cell (HSC) fate determination through the control of metabolic and epigenetic modifications [43, 44], again, in part linked to mitochondrial turnover. HSCs isolated from aged mice are reported to form distinct populations based upon autophagy activity, with approximately a third maintaining high autophagy levels and a similar regenerative capacity to HSCs isolated from young mice. In contrast, the remaining autophagy low, aged HSCs displayed a reduced regenerative capacity and a pro‐myeloid skewing, phenotypes characterizing aged blood. In support of these findings the loss of autophagy in genetically engineered mouse models is associated with the premature acquisition of aged blood phenotypes, including a myeloid skewing due to altered epigenetic priming of the HSCs [44]. Similarly, the loss of chaperone‐mediated autophagy with age is associated with a shift in fatty acid metabolism in HSCs and a loss of function, which can be reversed with pharmacological activators [45]. Such data suggest that the decline of autophagy with age may limit the capacity of stem cell populations to regenerate tissue damage, which over time will negatively impact both tissue structure and function.

Several of these studies have also provided evidence that boosting autophagy through pharmacological or genetic means can restore the functionality of these stem cell populations. This is in keeping with the myriad of papers that report positive health‐ and life‐span benefits through the pharmacological promotion of autophagy in mouse models, and suggests that autophagy may be a *bona fide* therapeutic target to extend organismal health span [5, 46]. This idea is further supported by genetic evidence from mouse models where autophagy has been constitutively promoted throughout life. Transgenic mice overexpressing Atg5 live longer, in part attributed to an enhanced metabolic state, including an improved insulin sensitivity and reduced adipose accumulation with age [19]. Life span is also extended in *Becn1* ^*F121A/F121A*^ knockin mice, in which a substitution mutation in the BH3 domain of Beclin1 increases autophagic flux by disrupting its interaction with the negative regulator Bcl2. This knockin allele also rescues premature aging in the *Klotho* hypomorphic mouse model [20]. However, it remains to be seen in these settings how tissue and stem cell populations change with increasing age, and whether these benefits are due to global autophagy promotion or the benefit can be attributed to alterations in tissue or cell‐type specific populations. Additionally, whilst boosting autophagic activity from birth appears to promote health and life span, this is almost certainly through prevention of macromolecular damage forming.

In support of the central role of autophagy in aging, whole‐body inhibition of autophagy (except for the brain) through the expression of a doxycycline‐inducible shRNA targeting Atg5 (Atg5i mice), also drives the development of macroscopic premature aging phenotypes such as graying, kyphosis, loss of muscle mass, and a shortening of life span in two closely related models [21]. These models differ in only their targeting sequence and the degree to which they inhibit autophagy; Atg5i mice provide a robust knockdown phenocopying a knockout in many tissues, meanwhile, Atg5i‐2 mice are hypomorphic, and despite a build‐up of p62/Sqstm1 lack the gross phenotypes associated with Atg5i and Atg5 knockout mice. Upon loss of autophagy markers of aging such as inflammatory cytokines, telomere‐associated DNA damage, and markers of senescence were also found elevated across various tissues [21]. When autophagy was restored, through doxycycline withdrawal and the re‐expression of Atg5, this led to a striking reacquisition of organismal health and life span. However, it should be noted that this rejuvenation is incomplete as evidence of molecular damage and markers of senescence remain elevated, whilst life span was still reduced in comparison to control mice (in which shAtg5 was not expressed). This suggests that some of the damage induced by loss of Atg5, and its resultant effects such as senescence induction, may be irreversible and might continue to contribute to organismal age‐related decline.

## 3 Autophagy and senescence

Cellular senescence is a distinct cellular state activated in response to stress (such as DNA damage, telomere attrition, mitochondrial dysfunction, and high oncogenic signaling), as well as present in normal physiological settings, such as during embryogenesis and wound healing [47, 48]. Phenotypically, it is characterized by a stable inhibition of proliferation, often accompanied by a high lysosomal mass (as shown by staining for senescence‐associated beta‐galactosidase activity, SA‐β‐gal), and the acquisition of a distinct secretory phenotype. This later feature, known as the senescence‐associated secretory phenotype (the SASP), is believed to underpin the functional role of senescence in diverse biology from biological patterning, wound healing, and immunosurveillance, to tumor promotion through the expression and release of a wide array of cytokines and signaling molecules that act both locally and systemically [49, 50, 51]. Due to its stable cell cycle arrest, senescence is considered to be a tumor‐suppressive program, and markers of senescence are often found upregulated in premalignant lesions [52]. Conversely, senescent cells through the SASP may also have deleterious effects through shaping the tumorigenic microenvironment and supporting tumor progression [53]. To understand the functional relevance of senescence *in vivo*, it is important to collectively consider these two aspects of senescence: stable exit from the cell cycle (cell‐autonomous), and the capability to modulate surrounding tissues (non‐cell‐autonomous).

### 3.1 Basal autophagy

While senescence, particularly that caused by telomere shortening, has been implicated in organismal aging, the causative relationship between these processes was not clear until relatively recently [54, 55]. A series of mouse studies in 2006 showed an age‐associated accumulation of cells expressing p16 (a cyclin‐dependent kinase inhibitor functionally linked to senescence) in HSCs, neural progenitors, and pancreatic β‐cells. Importantly, the decline of self‐renewal potential of these cells was in part dependent on p16 expression [56, 57, 58]. These studies collectively reinforced the idea that senescence in tissue stem cells or progenitor cells limits a tissue's regenerative capacity, thus enhancing age‐associated tissue degeneration [59]. This is reminiscent of the key role of autophagy in the maintenance of stem cell regenerative potential in HSCs and muscle satellite cells, as described above [43, 44, 60]. While senescence and autophagy have not been experimentally linked in HSCs in the context of aging, their functional association has been reported in muscle stem cells. In geriatric mice, the regenerative capacity of satellite cells declines partly through the development of senescence in these cells [60]. In a subsequent study, the same group showed that *Atg7* depletion in Pax7‐positive satellite cells induced senescence, and that geriatric senescence could be prevented in these cells by treating them with rapamycin or spermidine [41]. These results further indicate that an intimate relationship exists between organismal age, cumulative and degenerative alterations in organs, and the decline of cellular self‐renewal capacity, where basal autophagy actively serves to maintain cellular fitness, thus preventing senescence [53, 61].

In addition to the cell‐autonomous aspect of senescence, emerging evidence suggests its non‐cell‐autonomous activities contribute to aging. In an elegantly devised mouse model, in which p16‐expressing (and thus likely senescent) cells were induced to apoptose, the enforced elimination of these cells attenuated age‐associated deterioration in several organs in both progeroid mice (with a *BubR1* hypomorphic genetic background) and naturally aging mice, even in later life [62, 63]. While the exact mechanism of how senescent cells locally (in diverse albeit not all tissues) or even systemically promote the aging process remains to be elucidated, it has been attributed to the accumulating and persistent non‐cell‐autonomous activities of senescent cells, represented by the SASP [54, 55, 64]. How autophagy is involved in this scenario appears to be highly complex and is less well explored. However, as described below, abundant evidence indicates that senescence (both its cell‐autonomous and non‐cell‐autonomous aspects) and autophagy are stress‐induced processes.

### 3.2 Stress‐induced autophagy

Considering the role of basal autophagy in metabolic and energy homeostasis and in cytoprotection, it is not surprising that a reduction of basal autophagy induces some types of senescence, which itself is a highly heterogenous state [48, 53]. In addition to the *in vivo* studies described above (for example, in the Atg5i model and in satellite cells), autophagy inhibition has also been shown to promote senescence in cell culture models [65, 66]. But it remains unclear whether or not the age‐associated decline of autophagy activity is the major cause of an increased senescence load with age.

Autophagy is also activated in response to diverse cellular stress, including oncogenic stress and stress caused by chemotherapeutic reagents, both of which can also induce senescence. It was also shown in human fibroblasts that, during replicative senescence, induced through telomere attrition and resulting in persistent DNA damage response, there is an adaptive shift from the proteasomal system towards the autophagic pathway [67]. Indeed, the hallmark of senescence, SA‐β‐gal activity represents elevated lysosomal mass and function, likely to be reflecting autolysosomes at least in some contexts [68, 69, 70].

The functional relevance of autophagy in senescence appears to be highly diverse and context‐dependent. It is conceivable that autophagy activation may promote cell survival under stress conditions [71] but abundant evidence indicates that autophagy modulates specific senescence effectors at multiple levels, which are not necessarily linear. We have previously shown that autophagy is activated during human fibroblast senescence and that it promotes the SASP through mTOR signaling [70, 72]. While mTOR, which can promote the SASP at both post‐transcription (mRNA stability) and mRNA translation levels [73, 74] negatively controls autophagy upstream at autophagosome formation, amino acids, (outputs of autolysosomal degradation) activate mTOR at the surface of (auto)lysosomes [75, 76]. Interestingly, while starvation typically inhibits mTOR and activates autophagy, prolonged starvation reactivates mTOR through autolysosomal degradation products [77]. As such, a spatially regulated feedback module between mTOR and lysosomes coordinates anabolic (mTOR signaling) and catabolic (autophagy) response, allowing their simultaneous activation [70, 78].

Autophagy is also involved in modulating the senescence phenotype, including the SASP, through specific protein degradation. For example, Kang et al. [79] have reported that the transcription factor GATA4 is both a substrate of p62‐mediated selective autophagy and a positive regulator of the inflammatory SASP. Interestingly, they showed that transient autophagy inhibition, which stabilizes GATA4 and thereby promotes the SASP, induces senescence more efficiently compared to the continuous inhibition of autophagy, proposing that global and selective autophagy have opposite effects, promoting and mitigating senescence, respectively [79].

The selectivity of autophagy cargo also appears to alter during senescence. Lamin B1, a major component of the nuclear envelope, is downregulated during senescence, at least in part through selective autophagy degradation via APOE and LC3 binding [80, 81, 82]. Downregulation of Lamin B1, a widely recognized senescence feature [83, 84, 85, 86, 87, 88], has been functionally associated with other senescence features, including the formation of senescence‐associated heterochromatin foci (SAHFs) [87] and cytoplasmic chromatin fragments (CCFs) [82, 89]. CCFs are recognized by the cytosolic DNA‐sensing cGAS–STING pathway to activate the inflammatory SASP [90, 91, 92, 93]. In addition, a recent study identified that the cGAS–STING pathway can also activate autophagy in the context of replicative crisis, in which excessive autophagy can be detrimental [94]. Although these observations indicate a positive feedback loop between autophagy and the cGAS–STING pathway, autophagy can also degrade cytosolic DNA species [95, 96, 97], providing an additional layer of complexity in the SASP regulation and cellular viability.

Senescence‐associated nuclear lamina breakdown can be partly explained by a unique type of nuclear autophagy [98]. LC3 is a key protein involved in autophagy vesicle formation (thus often utilized as a marker of autophagosomes and autolysosomes puncta), but it also plays a role in the recruitment of autophagy cargos to autophagosomes [99, 100]. Interestingly, LC3 is also distributed in the nuclei and interacts with Lamin B1 at the basal state. During oncogene‐induced senescence in human fibroblast, the LC3‐Lamin B1 complex is recruited to autophagosomes and degraded at the cytoplasm [82]. This mechanism is not limited to Lamin B1. SIRT1, a conserved NAD+‐dependent deacetylase (substrates include histones and non‐histone proteins, such as p53 [101, 102, 103, 104], is involved in diverse biological processes, including senescence and aging [105, 106]. SIRT1 is known to be post‐transcriptionally downregulated during senescence, and its ectopic expression of SIRT1 delays replicative senescence in human fibroblasts [107, 108]. Similar to Lamin B1, Xu et al. [109] have shown that SIRT1 binds LC3 at the nuclei in a basal condition, and upon senescence induction, their interaction is enhanced and the complex is degraded in the cytoplasm through autophagy. This autophagy‐dependent SIRT1 downregulation was also seen in hematopoietic tissues during aging in both humans and mice [109]. LC3 has been previously shown to be a nuclear substrate of SIRT1: the deacetylation of LC3 promotes its exportation to the cytoplasm [110]. The study by Xu et al. is particularly relevant in senescence and aging as it indicates that SIRT1 is also a substrate of LC3‐mediated selective autophagy. Notably, SIRT1 appears to negatively regulate the expression of *IL6* and *IL8*, encoding major SASP factors, through direct binding to their promoter regions [111], providing an additional mechanistic link between autophagy and the SASP. Finally, the maintenance DNA methyltransferase, DNMT1 has also been shown to be degraded by autophagy during senescence induced by CDK inhibitors in cancer cells [112]. DNMT1 is also repressed during replicative senescence [113, 114], and that its depletion accelerates senescence in human fibroblasts [113].

**Fig. 2:**
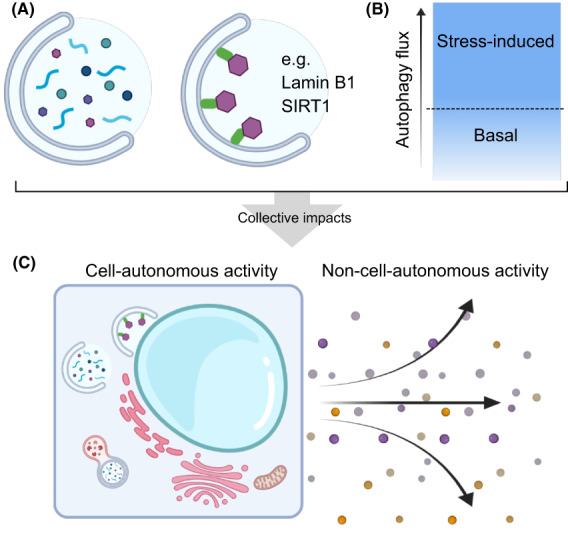
Basal and stress‐induced autophagy in senescence. (A) The collective effect of autophagy is both the bulk selective and nonselective degradation of cellular components. (B) During stress induction which may result in senescence, such as telomere attrition or oncogene activation, autophagy can be up‐regulated to maintain the cell‐autonomous and the non‐cell‐autonomous functionalities of a cell (as shown in C), i.e., proliferative arrest, metabolism, secretory outputs.

Combined, autophagy activation can modulate the phenotype through multiple paths. To understand the phenotypic impact of autophagy, it is crucial to consider the balance between basal and stress‐induced autophagy as well as between bulk and selective autophagy (Fig. 2). Some of the specific nuclear autophagy cargos are involved in the control of both epigenetic and secretory programs during senescence (e.g., Lamin B1 and SIRT1), reinforcing the idea that autophagy is a focal point on which cell‐autonomous and non‐cell‐autonomous regulation of senescence converges.

## 4 Autophagy and tumorigenesis

**Fig. 3:**
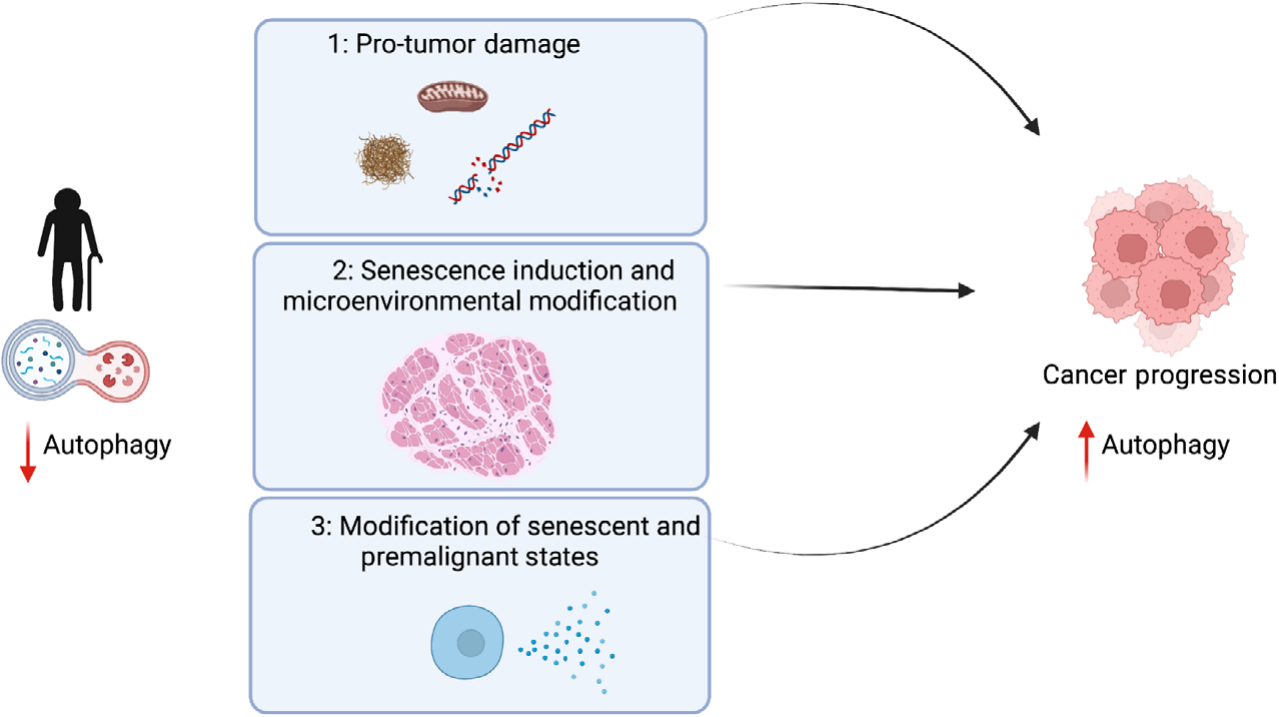
Dynamic fluctuations in autophagy promote cancer progression. With an increasing age and a subsequent decrease in autophagic flux, early tumor development can be promoted through several mechanisms. (1) Through the creation of pro‐tumor damage at the level of protein, organelle homeostasis, and/or DNA damage; (2) the induction of cellular senescence which can generate a pro‐tumorigenic microenvironment; (3) modification of precancerous cellular states accelerating the transition to malignant cancer.

During tumor development, the role of autophagy is complex and appears dependent upon the stage and tissue of origin. On one hand, autophagy is considered essential for cellular health and its loss may otherwise lead to the accumulation of pro‐tumor damage; as such, its promotion is believed to help prevent the development of this initial damage. Consistently, increased basal autophagy in *Becn1* ^*F121A/F121A*^ knockin mice not only extends lifespan but also diminishes age‐associated spontaneous tumorigenesis in comparison to littermate controls [20]. Meanwhile, as tumor formation is a microcosm of evolution, and with autophagy being a pro‐fitness and cytoprotective program, many end‐stage tumors are believed to be dependent on an intact, or enhanced autophagy process. As such dynamic fluctuations in autophagy may occur during the progression of the disease (Fig. 3).

In the liver, the accumulation of the autophagy receptor Sqstm1/p62 is considered a key initiating event in the development of malignant hepatocellular carcinoma [115]. Congruently, the loss of autophagy in either mice with whole liver deletion of Atg7 (*Atg7* ^*flox/flox*^; *Alb‐Cre*) or whole‐body mosaic deletion of Atg5 (*Atg5* ^*f/f*^; *CAG‐Cre*) results in hepatic adenoma development, and loss of Sqstm1/p62 severely impedes this [116]. Similarly, the loss of chaperone‐mediated autophagy through *Lamp2A* knockout (*Lamp2A* ^*flox/flox*^; *Alb‐Cre*) also promotes the development of hepatocellular adenomas with increasing age [117]. Yet whilst the loss of autophagic activity can drive the initial formation of tumorigenesis, this is limited to benign adenomas, and no malignant tumors arise [116].

Similarly, in lung models of autophagy inhibition, the loss of either Atg7 or Atg5 appears to significantly impact the development of aggressive disease in K‐ras^G12D^ driven models, yet with some notable differences. In the case of Atg7 knockout, tumors appear to develop at a similar rate in the early stages of disease regardless of autophagy status. However, whilst autophagy competent tumors develop into malignant tumors, Atg7 deficiency forces tumors to develop towards benign oncocytomas [118]. Of note, concomitant loss of Lkb1 with K‐ras^G12D^ activation is associated with a requirement for an intact autophagy system, and its loss severely impacts tumorigenesis, suggesting tumor genotype and timing of mutational acquisition may also dictate the role of autophagy in disease progression [119]. In the Atg5 knockout model, the loss of autophagy reduces tumor burden and progression to adenocarcinoma in the lung. Interestingly, the authors also noted that the initial transition from hyperplasia to adenoma in autophagy‐deficient mice was higher, suggesting that the loss of Atg5 in this model promotes the early stages of tumor development, whilst blocking the later stages [120]. In keeping with this, in K‐ras^G12D^ driven models of pancreatic cancer development, autophagy inhibition (via simultaneous deletion of Atg7 or Atg5) has shown to increase the incidence of early premalignant lesion development [121, 122]. However, despite this increase in early grade pancreatic intraepithelial neoplasia (PanIN) lesions, these rarely transition to malignant tumors except in the p53 knockout background.

Combined these data suggest that the loss of autophagy may provide initial evolutionary momentum, yet its continued absence is either insufficient alone to promote, or indeed may be detrimental in of itself to malignant conversion. This idea was tested in the Atg5i mouse model, in which autophagy can be dynamically regulated through doxycycline‐inducible expression of sh‐Atg5. Atg5i mice in which autophagy was inhibited for ~ 4 months displayed signs of premature aging with a reduction in health status. When autophagy was subsequently re‐established in these mice, these phenotypes were rescued as was their life span [21]. However, these mice also displayed an increase in age‐associated spontaneous tumor formation in comparison with control mice (where autophagy was intact all the time), thus transient systemic inhibition of autophagy appears to increase the tumorigenic risk. Of note, continuous autophagy inhibition in Atg5i mice was not associated with tumor development, although their premature death due to accelerated aging precluded this assessment. Nevertheless, given that malignant tumors failed to form in long‐lived mosaic Atg5 knockout mice [116], the transient inhibition of autophagy appears to increase the risk of cancer relative to the continuous inhibition of autophagy.

These findings support the theory that autophagy has phase‐dependent roles during tumor development, but what is the mechanism for ‘rejuvenation‐induced’ tumorigenesis, as seen in the Atg5i mice? It is conceivable that reduced autophagy leads to damage accumulation, either proteomic or (epi)genomic, which can be pro‐tumorigenic. Yet without sufficient autophagy, cellular fitness is below the threshold required to support malignant transformation. This idea is largely biased towards the cell‐autonomous aspect of the tumor origin. Notably, it has been shown that short‐term autophagy deficiency in mouse livers causes acute liver damage, which is mostly ‘cured’ by autophagy restoration [123]. However, this autophagy restoration also promotes liver fibrosis. Hepatic stellate cells are a major component of the fibrosis reaction, and autophagy inhibition prevents their function, as such the reactivation of autophagy may enable these cells remodel the tissue microenvironment [123, 124, 125]. Therefore, even though macroscopic restoration occurs in Atg5i mice, the local and tissue microenvironment could remain different. Indeed, as mentioned earlier, Atg5i mice also retain heightened levels of senescent cells after autophagy restoration. As such the outstanding question is whether those residual senescent cells support and shape tumorigenic microenvironment and/or can those pretumoral senescent cells with persistent DNA damage somehow occasionally regain the proliferative capacity with increased cellular fitness after autophagy restoration.

It is also clear that microenvironmental autophagy has an important role in end‐stage tumors as well as potentially during the initial development of cancer. In pancreatic cancer, it is reported that tumor cells promote autophagy in pancreatic stellate cells, which in turn supports tumor growth through metabolic support [126]. Similarly autophagy loss in hepatic stellate cells appears to reduced hepatocellular carcinoma growth, partly through loss of GDF15 expression [127]. Furthermore, several reports have also implicated hepatic autophagy in modulating systemic effects of tumor growth, either through maintaining circulating amino acid (arginine) [128] or inflammatory cytokine levels [129]. However, as these effects may be due to the dramatic effect of complete autophagy loss in the liver, exemplified by gross hepatomegaly and widespread hepatocyte cell death, it is not known to what degree these effects may be recapitulated during aging, when autophagic activity is decreased but not completely abrogated.

In this vein it should also be noted that the complete knockout models of autophagy represent extreme examples that do not fully recapitulate the natural biogenesis of disease states. Interestingly, human cancers are not associated with biallelic inactivating mutations in genes essential for autophagic activity. For example, early reports have described enhanced spontaneous tumor formation in mice with only one functional copy of the *Beclin1* gene [130, 131], which is often lost in a heterozygous manner in human breast, ovarian, and prostate cancer [132, 133, 134]. Although the proximity of *Beclin1* to the *BRCA1* tumor suppressor gene renders the interpretation difficult particularly in these tumor types [135], such a haplo‐insufficient tumor‐suppressive activity was also observed in the *Atg5*+/− mouse models for pancreatic cancer [136] and AML [137].

As such we must assume that, whilst our current genetic mouse models lend important insights into disease progression, more nuanced models in which autophagic activity (or even adaptor proteins and cargo) can be titrated, and do not profoundly introduce tissue architecture artifacts, are required. As examples, autophagy deletion in the liver is associated with extreme hepatomegaly and liver damage [123], whilst in the pancreas, its loss is associated with the development of pancreatic atrophy [121, 138]. Such phenotypes may impact the progression of disease, not necessarily in a way that mirrors the etiology of human disease with regards to autophagic activity and aging. In this regard, the Atg5i models are promising. Although the original Atg5i mice exhibited a highly efficient knockdown of Atg5, thus developing a phenotype reminiscent of complete knockouts, the second Atg5i model (Atg5i‐2), in which Atg5 is downregulated using a weaker shRNA and which produces milder phenotype with, e.g., no obvious hepatomegaly [21], still displays premature aging phenotypes. However, tumor susceptibility in the Atg5i‐2 remains to be evaluated and this model may be of greater interest in future studies. Similarly, the recently developed *HyD‐LIR* ^*flox/flox*^ mouse model, wherein Atg8‐dependent selective autophagy can be impaired, due to expression of a synthetic gene that acts as a competitive inhibitor to p62/sqstm1, offers a remarkable opportunity to study aspects of autophagy loss without the caveats that accompany the complete knockout of essential autophagy genes [139, 140].

## 5 Perspectives and conclusions

Advancing age represents the single greatest risk factor for cancer development. As organisms age, the rate at which autophagy occurs decreases. Indeed, it is clear that the loss of autophagy can lead to cellular dysfunction and can perturb stem cell populations *in vivo*, limiting their regenerative potential and reducing the homeostatic capacity of a tissue [41, 44]. As autophagy is associated with metabolism, mitochondrial homeostasis, reactive oxygen species generation, turnover of toxic and tumor‐promoting aggregates, as well as homeostasis of other organelles, it is likely that this age‐associated decrease in autophagy impacts some or all of these processes at once. However, to what degree each is compromised during aging and is functionally relevant to age‐associated decline is difficult to disentangle, and almost certainly cell‐type‐specific.

Importantly, the loss of basal autophagy appears also to be associated with the acquisition of cellular senescence [65, 141]. As mammals age, and concomitant with a decrease in autophagy, there is also an increase in the senescent burden, or the amount of senescent cells residing in tissues. The historical viewpoint, that senescence is primarily a cell autonomous process, has long since been expanded by the realization that senescent cells modify their surrounding local microenvironment, and have systemic effects on an organism [50, 53]. Yet whether the loss of autophagy is a major driver of senescence in naturally aged systems, or is limited to particular populations (i.e., stem cells with low proliferative indexes) remains to be investigated, as does the phenotype of this form of senescence. It is now widely accepted that senescence itself is a heterogenous cellular state that may depend on the cell of origin, and on the type of stress that led to its induction. As autophagy has been linked to the maintenance of senescence, metabolism, and secretion, it is tempting to speculate that this form of senescence will be distinct with unique effects on the local milieu [65, 70, 72, 142].

Senescence is also considered to be a barrier to tumorigenesis in a number of settings, and senescence markers are often found to be upregulated during the premalignant stages of cancer development *in vivo* [52]. As such the intersection of cancer development, particularly at these early stages of tumor formation, with an age‐associated decrease in autophagy represents a potential source of synergy for tumor promotion. How a reduction in autophagy, as opposed to its complete loss, in these early lesions may impinge upon senescent and premalignant biology is still relatively unexplored *in vivo*. Moreover, reduced levels of autophagy may lead to tumor progression through cell autonomous or nonautonomous mechanisms. In addition to its typical nonselective degradation, cargo selectivity also plays a key role in downstream phenotypes, such as stress‐induced senescence [72, 80, 82]. Selective autophagy, as exemplified by nuclear autophagy, appears to have a distinct layer of regulation, and the net effect of the bulk autophagy and fine‐tuned selective autophagy not only shape the intrinsic cellular phenotype but also surrounding tissue microenvironments by modulating non‐cell‐autonomous activities (e.g., the SASP). To‐date, only a handful of specific (nuclear autophagy) cargos that are associated with senescence have been characterized. The future identification of such cargos would enrich our understanding of the crosstalk between senescence and autophagy and their relevance to aging and cancer, and might provide new opportunities to intervene in aging and age‐associated disorders.

The promotion of autophagy throughout life has been shown to extend life and health span, with a reduction in spontaneous tumor formation in mammalian systems [20]. Whilst the genetic promotion of autophagy can restore cellular and tissue functionality, as well as extend or recapture health and life span, there is evidence that damage remains, including senescent cells, and may continue to have detrimental effects [19, 20, 21, 123]. Indeed, ‘rejuvenation’ via the re‐establishment of autophagy in Atg5i mice is accompanied by increased tumorigenesis in the presence of residual senescence cells. These persistent senescent cells may represent early tumor formation, or may promote tumor development through non‐cell autonomous activities. To‐date therapies targeting senescent cells for destruction with senolytics and therapeutics‐boosting autophagy have only been considered individually. However, this raises the question of whether senolytic compounds may synergize with autophagy augmentation to promote a more holistic organismal rejuvenation therapy.

## Conflict of interest

The authors declare no conflict of interest.
